# Whipple disease diagnosed by enteroscopy: first case report in Colombia of an underdiagnosed disease and literature review

**DOI:** 10.1186/s12876-020-01302-2

**Published:** 2020-06-23

**Authors:** Margarita Rey R., Luis Alejandro Orozco, Katherine Marrugo, Rocio López, Erika D. Pérez-Riveros, José De la Hoz-Valle, Fernando Sierra-Arango

**Affiliations:** 1 Gastroenterology and Hepatology Department, Digestive Endoscopy Section, Fundación Santa Fe de Bogotá, Cogotá D.C., Colombia; 2 Global Medical Center, Bogotá D.C., Colombia; 3 Universidad del Rosario, Bogotá D.C., Colombia; 4 Pathology Department, Fundación Santa Fe de Bogotá, Cogotá D.C., Colombia; 5 Subdirección de Estudios Clínicos y Epidemiología Clínica (SECEC), Fundación Santa Fe de Bogotá, Bogotá D. C., Colombia; 6 Gastroenterology and Hepatology Department, Digestive Endoscopy Section, Fundación Santa Fe de Bogotá and Universidad de los Andes Medical School, Bogotá D.C., Colombia

**Keywords:** Whipple disease, Tropheryma, Capsule endoscopes, Periodic acid-Schiff reaction, Antibiotics, Case report

## Abstract

**Background:**

Whipple’s disease is a rare systemic disease caused by a gram-positive bacillus called Tropheryma whipplei. First described in 1907 as an intestinal lipodystrophy with histological finding of vacuoles in the macrophages of the intestinal mucous. Usually the symptoms are localized according to the compromised organ. The differential diagnosis is wide. It can be fatal without proper treatment. Recurrence can occur in up to 33% of the cases and usually compromises the neurological system.

**Case presentation:**

This article reports the case of a 46-year-old female patient with a history of a 6-month hypochromic microcytic anemia of unknown cause. She consulted for a 6-months oppressive abdominal pain located in the mesogastrium as well as abdominal distention associated with nausea and liquid stools; in addition, she had an 8-month small and medium joint pain, without edema or erythema. Physical examination without relevant findings. Multiple esophagogastroduodenoscopies with normal gastric and duodenal biopsies findings and a normal colonoscopy were performed. Endoscope capsule showed red spots in the duodenum and ulcerations in the jejunum and proximal ileum covered by fibrin; histological report showed macrophages with positive periodic acid-schiff reaction staining (PAS staining), disgnosing Whipple’s disease. Antibiotics were initiated. The patient is currently in the second phase of treatment without gastrointestinal and joint symptoms.

**Conclusion:**

This is the first case reported in Colombia. It is a rare entity and difficult to diagnose reason why it is important to continue with clinical investigations to give more clarity about the onset and appropriate diagnose to avoid the delay in treatment of this entity.

## Introduction

Whipple’s disease (WD) was first described by George Hoyt Whipple in 1907 as an intestinal lipodystrophy [1]. It is characterized by the histological finding of vacuoles in the macrophages of the intestinal mucous in affected patients [2]. In 1990 a gram-positive bacterium causing this entity was identified, it amplified RNA segments by polymerase chain reaction (PCR); they called it Tropheryma whipplei (from the Greek trophe which means nutrition and erima that means barrier, given the result of malabsorption) [3]. According to literature, the most common host of this bacterium is the mucous of the small intestine in humans [4]. Once in the intestine the bacteria are phagocytosed by macrophages, where it can replicate [5]. This is a rare disease, the annual incidence of this entity has been 12 cases per year worldwide [1]. It is more common among farmers, sewer workers, people working with plants and soil or exposed to animal feces [6]. It is more common in middle-aged Caucasian men, suggesting there is a genetic predisposition for this disease [7]. Martinetti et al found HLA-DRB1^13 and DQB1^06 alleles could be a risk factor to consider in patients with WD [8]. However, immune response of the host has been evaluated, suggesting the symptoms vary depending on if the host has an immune deficiency [9].

Clinically it has two stages: 1) initial or prodromal; 2) stationary or latent. The first stage is characterized by compromising the joints. The second stage is characterized by weight loss, diarrhea and localized symptoms depending on the compromised organ. It can also be asymptomatic, characterized by being a carrier of the bacteria, finding it in feces and saliva; acute or chronic [10]. The differential diagnosis is wide; it includes other infectious causes, inflammatory rheumatic disease, malabsorption with compromise of the small intestine (celiac disease, sarcoidosis and lymphoma) and connective tissue diseases [11]. This is a disease that can be fatal without proper treatment [10]. Recurrence can occur in up to 33% of the cases and it usually compromises the neurological system [12]. In Colombia there are no cases reported. This is a rare entity and difficult to diagnose given the diversity of the clinical manifestations according to the organ affected [11]. This article reports the case of a patient with chronic diarrhea, anemia and arthralgias; a patient that required a complete examination of the small intestine and histopathological studies to make the diagnosis of Whipple’s disease and initiate adequate treatment. The aim of this report is to make a short literature review of this disease, given the importance of appropriate diagnostic methods and an on-time treatment.

## Case report

A 46-year-old female patient with history of a 6-month hypochromic microcytic anemia of unknown cause, controlled hypothyroidism and breast cancer 11 years ago that required quadrantectomy and radiotherapy, currently in remission. She consulted for a 6 month oppressive abdominal pain located in the mesogastrium, additionally she had abdominal distention associated with nausea and liquid stools (without mucous nor blood), approximately 4–5 stools per day; denying weight loss or fever.

The patient mentioned that 10 months ago she had traveled to Egypt and that for 8 months she had had joint pain (medium and small), without edema or erythema. She denies any history of allergic, toxic or transfusion events; only mentioning a family history of rheumatoid arthritis. During her physical examination she was clinically stable with vital signs in normal ranges, no relevant findings were found. Lab results: Hemoglobin 12.2 g/Dl, Hematocrit 38.4% MCV 76,3 MCH 22.9, normal hepatic function, TSH 1.5 UI/mL, negative Antinuclear antibodies, Rheumatoid factor, anti-DNA and IgA anti-transglutaminase antibodies. Negative serial coproscopic and coproculture. IgA levels in normal ranges. Normal abdomen tomography.

Given the presence of chronic diarrhea, studies are initiated to rule out infectious causes; tests for celiac disease were negative. Physicians performed endoscopic studies: multiple esophagogastroduodenoscopies up to the second duodenal portion evidenced an erythematous pangastropathy with normal findings in the gastric biopsies (two of these had duodenal biopsies with normal findings), and a colonoscopy with normal-looking mucous.

**Fig. 1:**
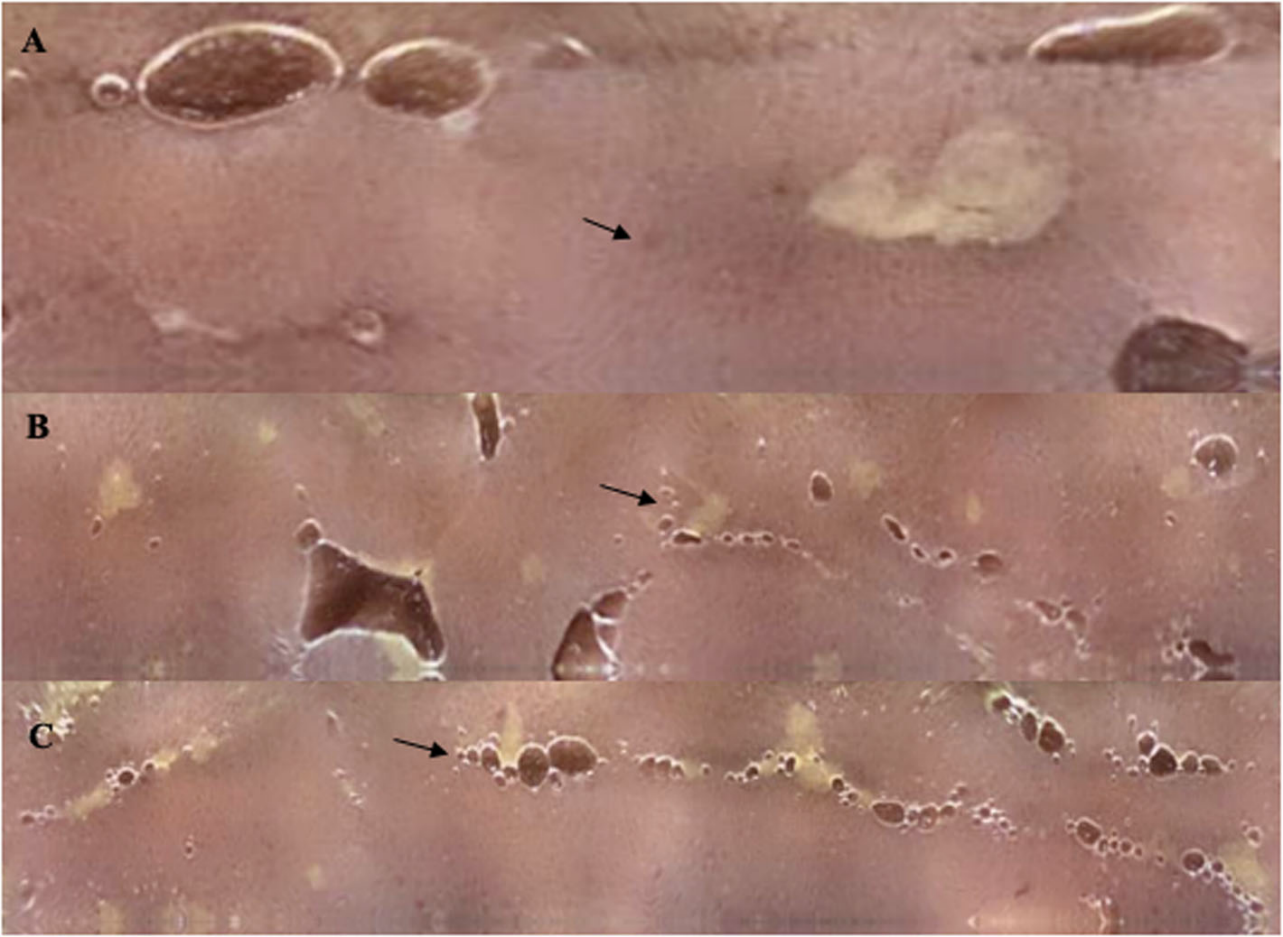
Endoscope capsule findings: **a** Shows red spots in the duodenum (indicated by the black arrow). (**b**-**c**) Shows multiple ulcerations in the jejunum and proximal ileum covered by fibrin (indicated by the black arrows)

**Fig. 2:**
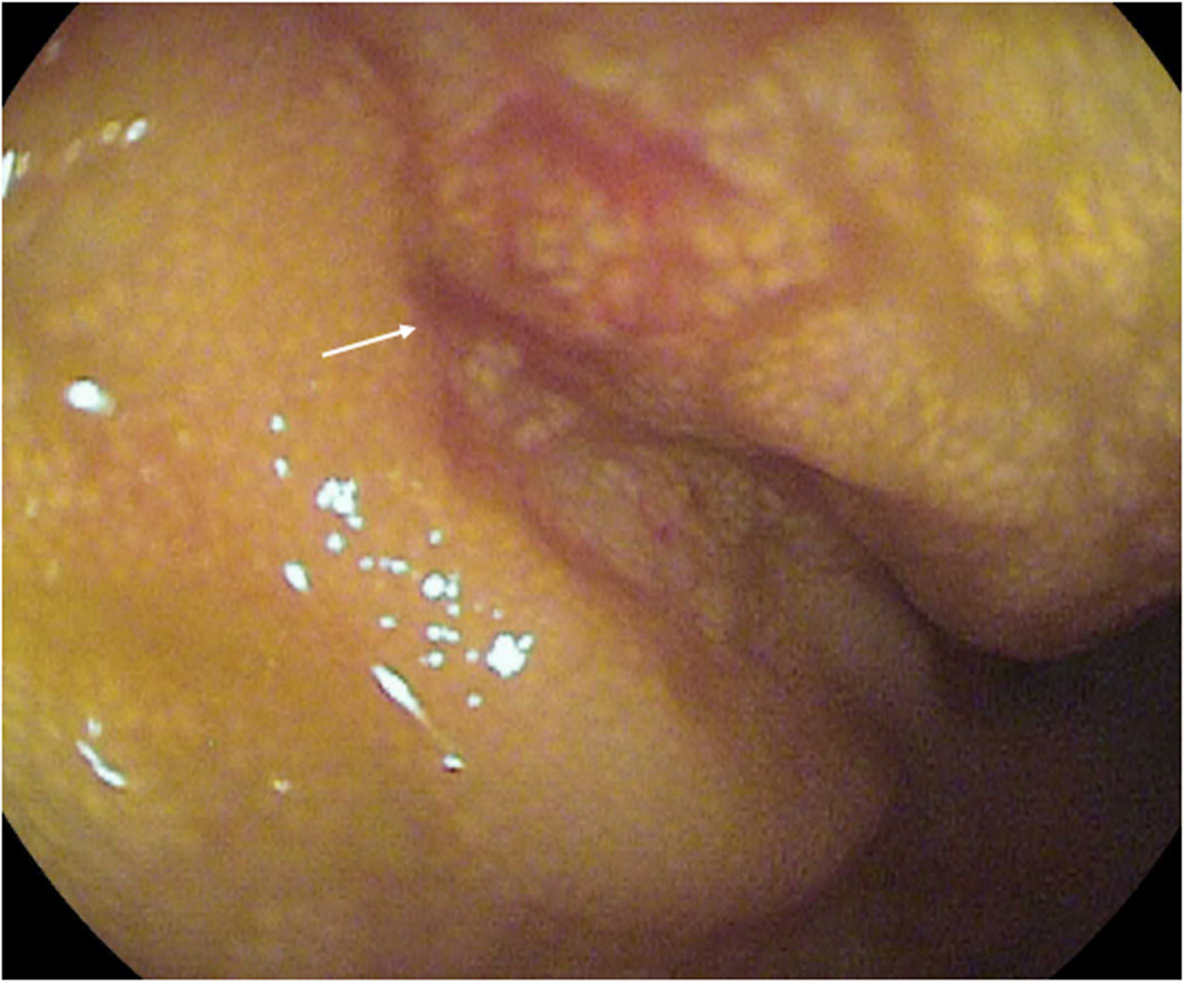
Enteroscopy: Showing an atrophic mucous throughout the path of the small intestine next to the ileocecal valve. Small non-aphtoid ulcers (indicated by the white arrow)

Due to the persistence of gastrointestinal symptoms and the presence of anemia, physicians decided to perform an endoscope capsule, showing red spots in the duodenum and multiple ulcerations in the jejunum and proximal ileum covered by fibrin (Fig. 1). A double balloon enteroscopy was performed showing an atrophic mucous throughout the path of the small intestine (Fig. 2); next to the ileocecal valve, there were small aphtoid ulcers. The jejunum and proximal ileum histological report showed macrophages with positive periodic acid-schiff reaction staining (PAS staining), being able to diagnose Whipple’s disease.

Antibiotics were initiated in 2 phases: Intravenous Ceftriaxone 1 g bid for 30 days; and at-home treatment with Trimethoprim / Sulfamethoxazole 160 mg/800 mg bid for 12 months. The patient is currently in the second phase of treatment with improvement of gastrointestinal and joint symptoms.

## Discussion

Whipple’s disease is a rare multisystemic disease that usually involves the small intestine, caused by a gram-positive bacillus. It is important to consider this entity in people with recent trips to endemic areas where polluted water acts as a possible source of infection [6]. Other predisposing factors are immunosuppression and human immunodeficiency virus (HIV) positive patients [9]. In this case, the patient had two high risk factors: a trip and her immunosuppression history prior to presenting symptoms. The classic clinical manifestation affects the gastrointestinal tract (abdominal pain, malabsorptive diarrhea and weight loss) or the musculoskeletal system (seronegative arthropathy with predominantly small joint migratory characteristics); low grade fever, anemia and adenopathy’s are other less common symptoms along with neurological, cardiac and pulmonary involvement [13].

Once the disease is suspected, it is important to rule out other differential diagnoses such as inflammatory bowel disease, other infectious causes, connective tissue diseases, immunosuppression and hyperthyroidism [13]. The diagnosis is usually made with endoscopic procedures and biopsies of the affected tissue. Whipple lesions are commonly described as pale-yellow shaggy mucosa in the duodenum and/or jejunum; also described as whitish-yellow plaques in a patchy distribution [14]. However, in 30% of cases the duodenal biopsies can be normal. This is why complete exploration of the small intestine may be necessary in some cases. Capsule endoscope is a non-invasive diagnostic tool that allows an appropriate study of the small intestine characterized by villous atrophy, erosions, lymphatic dilation, areas of denuded mucosa and especially diffuse whitish stippling [15]. Thus, in the literature there are scarce cases described where the diagnose was made with capsule endoscope, in this case it had a key role to achieve the diagnosis, since both upper and lower digestive endoscopy were inconclusive and the initial duodenal biopsies were non-diagnostic.

Certain methods available for diagnosis are: PCR and immunohistochemistry; in this test macrophages are detected with large amounts of positive PAS particles resistant to diastase in the lamina propria. In patients who do not have gastrointestinal symptoms, samples are taken from clinically compromised sites [16]. The sensitivity of the PAS staining of small bowel biopsies ranges from 71 to 78%. In some cases, PCR is needed to confirm the diagnose given its higher sensitivity and specificity, however PCR is not available in all centers. The final diagnosis is made if it meets one of the following criteria: a positive PAS staining in duodenal tissue, two different positive tests of the same tissue or two positive tests of different tissues [17].

When the diagnosis is confirmed it is recommended to perform PCR in the cerebrospinal fluid to rule out infection in the central nervous system; up to 50% of the patients with neurological impairment are asymptomatic. In other cases, the neurological symptoms are irreversible, leaving important sequelae in the patient. Given CNS symptoms appear in up to 15% of the cases with or without gastrointestinal manifestations, it is important to cover CNS to avoid catastrophic complications. This is why antibiotics used for treatment must cross the blood-brain barrier [14].

The recommended gold standard treatment is with Trimethoprim-sulfamethoxazole 160/800 mg orally twice daily for a least 1 year. Preceded by a 2-week parenteral therapy of ceftriaxone (2 g per day) able to penetrate the blood-brain barrier. There are different alternative treatments, depending in the toxicity and tolerance of the patients to certain antibiotics. Clinical improvement is beginning to be evident from days 7–21. Patients may relapse in up to 20% of cases [12].

The risk of relapse is unknown; these are largely responsible for a higher morbid-mortality, meaning the prognosis worsens with relapse and it commonly occurs after treatment; however, patients can present a relapse up to 30 years after the treatment stopped [18]. Saito et al. [19], performed repeated gastrointestinal endoscopy yearly as a follow up test to the patients treatment, to confirm the changes in the duodenal mucosa. He confirmed a complete recovery after a 2-year treatment, with rapid improvement in symptoms and endoscopic findings in the early stages of the treatment; the patient has been symptom-free for 8 years. In this case our patient was treated with the classical 1-year treatment and responded adequately to the pharmacological management. She is in the long-term second phase therapy. The follow-up to evaluate the clinical evolution was done. As soon as the patient finishes the treatment, a control capsule endoscope will be performed to evaluate the evolution and improvement of the mucosa.

## Conclusion

Whipple’s disease is a rare multisystem disorder whose main involvement is located in the small bowel. There are multiple ways to study this entity since the risk of sequelae and mortality rate are high if it’s not diagnosed and treated in a timely manner. In Colombia there are no reported cases of this disease despite its probable appearance in some institutions. Therefore, this case is considered necessary and important for the contribution to the medical literature given the need to increase the knowledge of this entity. In the same manner, it is important to continue investigating and clarifying the appropriate diagnostic methods to avoid the risk of complications due to the delay in treatment of this entity. Follow-up studies are recommended to assess the long-term outcome of these patients.

## Data Availability

Data sharing is not applicable to this article as no datasets were generated or analyzed during the current study.

## Abbreviations

WD: Whipple’s disease
PCR: Polymerase Chain Reaction
PAS staining: Periodic Acid-Schiff Reaction staining
HIV: Human Immunodeficiency Virus
CNS: Central Nervous System
